# PROM and Labour Effects on Urinary Metabolome: A Pilot Study

**DOI:** 10.1155/2018/1042479

**Published:** 2018-02-04

**Authors:** Alessandra Meloni, Francesco Palmas, Luigi Barberini, Rossella Mereu, Sara Francesca Deiana, Maria Francesca Fais, Antonio Noto, Claudia Fattuoni, Michele Mussap, Antonio Ragusa, Angelica Dessì, Roberta Pintus, Vassilios Fanos, Gian Benedetto Melis

**Affiliations:** ^1^Department of Surgical Sciences, Division of Gynaecology and Obstetrics, University of Cagliari, Cagliari, Italy; ^2^Department of Chemical and Geological Sciences, University of Cagliari, Cagliari, Italy; ^3^Department of Medical Sciences and Public Health, University of Cagliari, Cagliari, Italy; ^4^Department of Surgical Sciences, Neonatal Intensive Care Unit, Puericulture Institute and Neonatal Section, Azienda Ospedaliera Universitaria, Cagliari, Italy; ^5^Laboratory Medicine Service, IRCCS AOU San Martino-IST, University-Hospital, Genoa, Italy; ^6^Maternal Neonatal Department, Division of Gynaecology and Obstetrics, Massa Carrara Hospital, Carrara, Italy

## Abstract

Since pathologies and complications occurring during pregnancy and/or during labour may cause adverse outcomes for both newborns and mothers, there is a growing interest in metabolomic applications on pregnancy investigation. In fact, metabolomics has proved to be an efficient strategy for the description of several perinatal conditions. In particular, this study focuses on premature rupture of membranes (PROM) in pregnancy at term. For this project, urine samples were collected at three different clinical conditions: out of labour before PROM occurrence (Ph1), out of labour with PROM (Ph2), and during labour with PROM (Ph3). GC-MS analysis, followed by univariate and multivariate statistical analysis, was able to discriminate among the different classes, highlighting the metabolites most involved in the discrimination.

## 1. Introduction

The early diagnosis of pregnancy-related complications and the prediction of pregnancy outcome are considered strategic clinical goals to ensure the health of mothers and of their babies. Among these, premature rupture of membranes (PROM) consists of the rupture of the foetal membranes before the onset of labour. It can be observed at any gestational age [1] and occurs in approximately 10% of pregnant women and in roughly 40% of preterm deliveries [2]. Foetal membranes are of pivotal importance because they offer a robust barrier against infection ascending from the reproductive tract; after their rupture, both the mother and foetus are at risk of infection and other complications. The most commonly diagnosed maternal infections in case of PROM are chorioamnionitis and endometritis, which may be further promoted by frequent vaginal exams and the presence of meconium in the amniotic fluid (AF) [3]. Foetal complications of PROM include neonatal sepsis, abnormal foetal presentation, cord prolapse or compression, and abruptio placentae, and these may increase the risk of neonatal intraventricular haemorrhage, leading to neurodevelopmental disability as a direct consequence [4]. Therefore, from PROM, diagnosis may derive different medical procedures such as hospitalization, antibiotic prophylaxis, and induction of labour through oxytocin [5, 6], that is, increased medicalization and caesarean section rates. These practices may in turn affect several women in the positive experience of birth [7]. Moreover, given the increasing antibiotic resistance, alerts have been issued about the use and abuse of prophylactic antibiotic administration [8]. Indeed, growing evidences on this phenomenon suggest possible short- and long-term risks on maternal and foetal microbiota, resulting in long-term sequelae such as obesity, food allergies and intolerances, autoimmune diseases, and possible neurodevelopmental involvement. Although the exact aetiology is unclear, known factors are collagen remodelling, apoptosis [9], increased transcription of matrix metalloproteinases (MMP) such as MMP9, AF apoptotic activators [10, 11], and polymorphism promoter of tumour necrosis factor *α* (TNF-α), interleukin-1 (IL-1), and MMP1 [12, 13]. Notably, there is no universally accepted method for the diagnosis of PROM. Strategies currently performed consist of sterile speculum examination and nitrazine or fern tests [14], while ultrasound is useful to identify an AF reduction in the case of suspect membrane rupture [15]. Albeit these techniques have been employed for more than 60 years, the nitrazine test has been recently discouraged [16]. Moreover, since the AF concentration of several biomarkers is higher than that in normal vaginal secretion, many studies investigated the diagnostic value of vaginal AF for an early and accurate diagnosis of PROM. As a result, a number of potential biomarkers including prolactin, *α*-fetoprotein (AFP), *β*-subunit of human chorionic gonadotropin (*β*-HCG), foetal fibronectin, diamine oxidase, lactate, creatinine, urea, and insulin growth factor-binding protein-1 (IGF-BP1) have been proposed and tested [17, 18]. In particular, IGF-BP1 is the major protein in AF, and its presence confirms AF contamination in vaginal secretions. Interestingly, most of these biomarkers seem to accurately distinguish patients with intact membranes from those with unequivocal membrane rupture; however, they are not routinely applied due to their complex procedure, cost, and low sensitivities in patients with equivocal rupture. For these reasons, further investigations are necessary for the development of novel, versatile, and timely accurate diagnostic means. Among the most recent methods of investigation, metabolomics was successfully applied to describe the different molecular profiles arising over gestation [19] as well as the dynamics responsible for maternal and foetal unfavourable outcomes and labour and delivery complications [20, 21]. As a matter of fact, characterisation of the metabolic profile in various biological fluids such as AF, urine, maternal and cord blood, and vaginal secretions is considered one of the most promising and attractive tools for an early and accurate identification of several maternal morbidities and childbirth events [22]. Therefore, the present work applied a metabolomic approach to investigate the urinary metabolome in relation to PROM occurrence and labour. To pursue this goal, metabolic differences were observed in women with intact membranes and out of labour, with PROM and prior to labour, and with PROM and during labour.

## 2. Materials and Methods

### 2.1. Study Design and Population

Between October 2013 and July 2014, thirty-eight pregnant women at term, age 29–42 years (gestational age (GA) between 38 weeks + 0 day and 40 weeks + 4 days) with single, low-risk pregnancy, and foetuses in vertex presentation were admitted at the Unit of Obstetrics and Gynaecology of the University-Hospital of Cagliari and enrolled in this study. Patients gave written informed consent at the time of admission. The study was conducted in accordance with the Declaration of Helsinki (1964) and previously approved by the local ethics committee. Women were divided into 3 phenotypical groups. The first phenotypical group Ph1 consisted of 11 healthy pregnant women enrolled long time before labour (out of labour and intact membranes), these women were successively admitted with PROM. Group Ph2 consisted of 10 pregnant women with PROM (out of labour and PROM). Group Ph3 consisted of 17 pregnant women with prior diagnosis of PROM and in labour (in labour and PROM). Diagnosis of PROM was based on women's history, direct visualization of fluid leakage, and speculum examination. When direct visualization of AF loss was unreliable, a qualitative immunochromatographic dipstick test for AF IGF-BP1 presence, together with ultrasound AF evaluation, was performed. Diagnosis was then retrospectively confirmed after delivery.

### 2.2. Sample Collection and Storage

A spot urine sample was collected from each pregnant woman enrolled in the study; hence, a total of 38 samples were collected and analysed. For the Ph1 group, the urine was collected 3–14 days before delivery (median value 7 days, interquartile range (IQR) 5–11 days); for group Ph2, 15–50 hours before delivery (median value 28.5 hours, IQR 25.5–40 hours); and for group Ph3, 0.25–19 hours before delivery (median value 10 hours, IQR 5–13 hours). Sampling was performed through a sterile, preservative-free urine beaker equipped with a transfer device (VACUETTE®, Greiner Bio-One International GmbH, Kremsmünster, Austria) which allowed for the automatic filling of a vacuum urine tube without any external contamination. In detail, urine passes from the beaker to the vacuum tube by pushing it into the transfer device. The tube is then automatically filled for about 10 mL. After collection, all tubes were centrifuged and the supernatant was immediately frozen and stored at −80°C until analysis.

### 2.3. Sample Preparation

Urine samples were treated as previously described [23]. In brief, specimens were thawed at room temperature, 150 *μ*L was transferred into Eppendorf tubes, and 800 *μ*L of urease solution (1 mg/mL) was added. Following 30-minute sonication and deproteinisation, samples were centrifuged at 14000 rpm and 1200 *μ*L of the supernatant was dried in a vacuum centrifuge overnight. 30 *μ*L of a 0.24 M solution of methoxylamine hydrochloride in pyridine was added to each vial and kept at room temperature for 17 hours. 30 *μ*L of *N*-methyl-*N*-trimethylsilyltrifluoroacetamide (MSTFA) was added and kept at room temperature for 1 hour. The derivatized samples were diluted with 600 *μ*L of a tetracosane solution in hexane (0.01 mg/mL) just before GC-MS analysis.

### 2.4. Sample Analysis and Data Processing

The derivatized samples were analysed by using a global unbiased mass spectrometry-based platform with GC-MS incorporating an Agilent 5975C interfaced to a GC 7820 (Agilent Technologies, Palo Alto, CA, USA). The system was equipped with a DB-5 ms column (Agilent J&W Scientific, Folsom, CA, USA); injection temperature was set at 230°C and detector temperature at 280°C. Carrier gas (helium) flow rate was equal to 1 mL/min. GC oven starting temperature programme was 90°C with 1 min hold time and ramping at a rate of 10°C per minute, reaching a final temperature of 270°C with 7 min hold time. 1 *μ*L of the derivatized sample was injected in split (1 : 20) mode. After a solvent delay of 3 min, mass spectra were acquired in full-scan mode using 2.28 scans per second, with a mass range of 50–700 Amu. Each acquired chromatogram was analysed by means of the free software Automated Mass Spectral Deconvolution and Identification System (AMDIS) (http://chemdata.nist.gov/mass-spc/amdis). Each peak was identified by comparing the corresponding mass spectra and retention times with those stored in an in-house library including 255 metabolites. Other metabolites were identified by using the National Institute of Standards and Technology's mass spectral database (NIST08) and Golm Metabolome Database (GMD; http://gmd.mpimp-golm.mpg.de/). Metabolites were considered positively identified with a match factor ≥ 70%. For lower values, metabolites were labelled as “unknown.” This analysis produced a matrix spreadsheet containing 84 metabolites, 77 identified and 7 unknown, to be submitted to chemometric analysis.

### 2.5. Statistical Analysis

Sample size was adequate to assure the minimum precision requested for a pilot study [24]. All analyses were performed on MetaboAnalyst 3.0 (http://www.metaboanalyst.ca/) [25]. Both univariate and multivariate approaches were applied. In particular, univariate analysis consisted in analysis of variance (ANOVA) with Tukey's honestly significant difference (HSD) post hoc test with a false discovery rate (FDR) cut-off of 0.05 for analysis on more than two phenotypes, while Student's *t*-test with *p* < 0.05 cut-off was conducted for 2-phenotype models. Multivariate analysis was performed by means of the supervised partial least squares discriminant analysis (PLS-DA) in order to identify important variables with discriminative power, named variable importance in projection (VIP), and their trends. PLS-DA models were then submitted to a 10-fold cross-validation (CV) method for the evaluation of statistical parameters (accuracy, *R*^2^, and *Q*^2^) and the determination of the number of components that best describe the models. The PLS-DA model was further validated by permutation tests based on prediction accuracy (*n* = 100 and *p* < 0.01). The power analysis test was performed to determine the sample size required to detect a statistically significant difference between the two populations with a given degree of confidence (FDR = 0.1).

## 3. Results and Discussion

### 3.1. Three-Phenotype Model

First, statistical analysis and comparisons were conducted on all 3 different phenotypes at the same time. Applying ANOVA with Tukey's HSD post hoc test on the three classes Ph1, Ph2, and Ph3 resulted in 58 significant metabolites. For discussion purpose, these compounds were divided into 5 groups corresponding to different chemical classes: carbohydrates (Carb), oxidised carbohydrates (Ox), amino acids (AA), sugar related (SR), and miscellaneous (Misc). Metabolites' list for such calculations is reported in Table 1.

**Figure 1:**
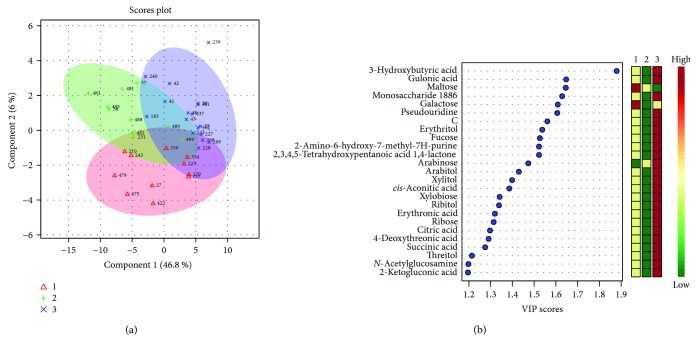
(a) 2D score plot showing PLS-DA discrimination between Ph1 (red, out of labour, intact membranes), Ph2 (green, out of labour, PROM), and Ph3 (blue, in labour, PROM) and (b) the corresponding VIP score plot.

PLS-DA of the same classes produced an unsatisfactory model scoring accuracy = 0.67, *R*^2^ = 0.88, and *Q*^2^ = 0.33. Moreover, the permutation test delivered a *p* = 0.04 (Figure 1)

### 3.2. PROM Model: Ph1 (Out of Labour, Intact Membranes) versus Ph2 (Out of Labour, PROM)

**Figure 2:**
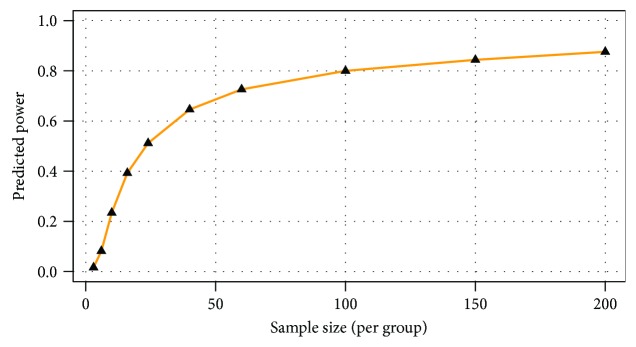
Power analysis calculations indicated a predictive power of 0.83 for 120 samples per group and an FDR of 0.1.

In order to investigate the metabolic differences caused solely by the rupture of membranes, the *t*-test was performed on Ph1 and Ph2 groups. This calculation highlighted 9 significant metabolites: galactose, uric acid, 3,4-dihydroxybutyric acid, galactitol, alanine, lysine, 4-hydroxyphenylacetic acid, serine, and hydroxyproline dipeptide. Notably, the entire set of metabolites resulted more abundant in Ph1, except for uric acid. This means that these metabolites are significantly consumed during PROM events. Also in this case, PLS-DA produced an unsuitable model with accuracy = 0.76, *R*^2^ = 0.43, *Q*^2^ = 0.29, and *p* = 0.09. However, the first 9 metabolites from PLS-DA correspond to those obtained from the *t*-test, and higher levels for all metabolites were observed in the Ph1 group. Power analysis calculated the number of samples for a predictive power of 0.83 as 120 per group (Figure 2).

### 3.3. Labour Model: Ph2 (Out of Labour, PROM) versus Ph3 (in Labour, PROM)

Labour effects on the metabolome were highlighted by the comparison of Ph2 and Ph3 groups. The *t*-test indicated 60 significant metabolites between the two groups of interest (Table 2).

**Figure 3:**
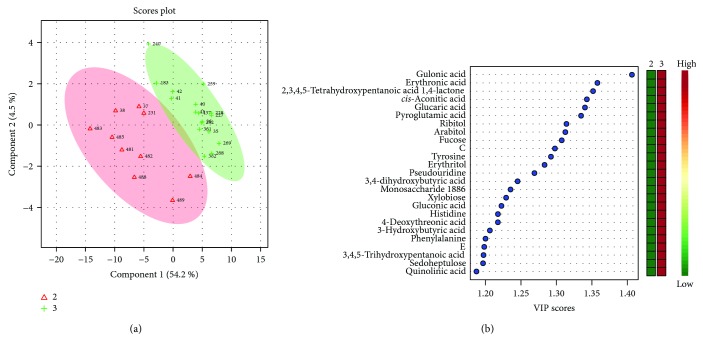
(a) Score plot showing the separation and clustering between Ph2 (2, triangles) and Ph3 (3, crosses) and (b) the corresponding VIP score plot.

Unsatisfactory PLS-DA results delivered a model with accuracy = 0.88, *R*^2^ = 0.96, *Q*^2^ = 0.54, and *p* = 0.04. Scores and VIP plots are shown in Figure 3.

**Figure 4:**
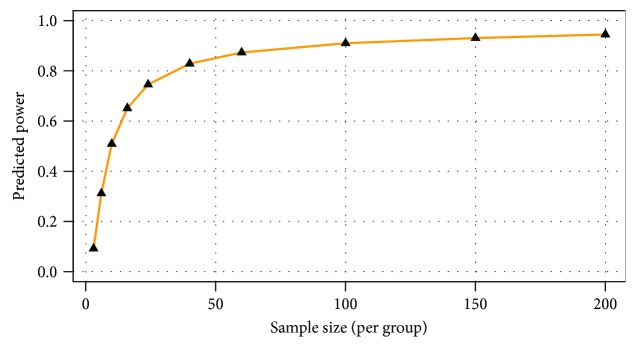
Power analysis calculations indicated a predictive power of 0.83 for 50 samples per group and an FDR of 0.1.

The majority of the metabolites responsible for such phenotype discrimination showed higher levels in the Ph3 group (in labour with PROM), while phosphate, lactose, and uric acid were more abundant in Ph2. Therefore, the metabolites are mainly produced during labour. Power analysis indicated better results for this model: a predictive power of 0.83 for 50 samples per group (Figure 4).

## 4. Discussion

Currently, assessment of PROM is mainly based on external genital leakage and/or direct observation of AF loss by direct visualization through speculum examination. Other options are biochemical tests and ultrasound AF evaluation, but none of these strategies may securely confirm diagnosis [26]; subsequently, confirmation of PROM often occurs during labour. Nevertheless, it is of crucial importance to make accurate and timely diagnosis in order to define appropriate clinical interventions, hence to avoid complications for the patients. In this study, although multivariate analysis could not reach statistical significance, univariate calculations identified several discriminant metabolites for the 3 phenotypes; 35 out of 58 could be distinguished between at least two comparisons. Therefore, the metabolic profiles are indeed altered due to PROM and/or labour. Nevertheless, more samples are necessary to provide a holistic model that may describe such heterogeneous and particular conditions. In particular, the separation between Ph2 and Ph3, which is characterised by PROM and differs on the onset of labour, seems to require the lowest number of samples and showed the highest number of discriminant metabolites through the *t*-test. For these reasons, it may be hypothesised that labour event affects more the system than PROM.

Furthermore, the fact that the PROM model (Ph1 versus Ph2) produced the lowest values for the statistical parameters may also suggest the eventuality that these two phenotypes are actually similar. Indeed, PROM per se may not represent a pathological event in the absence of complications. Therefore, further analysis should consider the outcome of delivery in PROM subjects. In detail, by analysing the alteration due to PROM, the almost totality of the discriminant compounds showed higher levels in the intact membrane group; hence, the discriminant compounds are significantly consumed in the case of broken membranes. Unfortunately, discriminant metabolites are of difficult interpretation. By observing the labour model (Ph2 versus Ph3), in labour subjects excreted higher amounts of the majority of the significant metabolites, while phosphate, lactose, and uric acid are excreted in lower amounts. Among the several significant metabolites, 3,4-dihydroxybutyric acid is a product of the oxidative metabolism of fatty acids and its increase can be observed in case of inflammations to satisfy the need for a surplus of energy due to stress conditions. Interestingly, it seems that inflammation processes may trigger preterm delivery [27]. Another sign of oxidative stress is the higher levels of glucuronic, gulonic, glucaric, and gluconic acids and other oxidised carbohydrates (Table 2) which derive from oxidative conversion [27, 28]. Moreover, the discriminant uric acid is involved in antioxidant activity in blood toward peroxyl radicals, which are released due to oxidative stress and ROS overproduction in labour [29]. By comparing the results from labour model calculations to those reported [30], only *cis*-aconitic acid showed an analogous trend. Indeed, this metabolite showed an upregulation characterising the active labour phase. *cis*-Aconitic acid is a well-known intermediate of the tricarboxylic acid (TCA) cycle, and its high level may be explained by the increased energy demand during labour.

## 5. Conclusions

GC-MS-based analysis of pregnant women's urinary metabolome could deliver interesting information about PROM and labour events. Most pregnant women at term will start labour in the next 24 hours after PROM; in fact, clinical guidelines recommend to wait at least 24 hours before labour induction. Intriguingly, further studies may investigate metabolic profiles associated with the timely spontaneous onset of labour to appropriately select women for labour induction.

Although the number of samples was not optimal for solid evidence, univariate chemometric analysis was able to discriminate among the different conditions, highlighting variation in the phenotypes' metabolome. An interesting feature of these data is that labour conditions seem to have greater influence on the system than the actual PROM occurrence, suggesting that further studies are in need for this delicate diagnosis. Nevertheless, a metabolomic approach on appropriate sample size may be a promising tool for research in the field of obstetrics.

## Figures and Tables

**Table 1:** Statistically significant (FDR < 0.05) metabolites from univariate analysis (ANOVA with Tukey's HSD post hoc test) of the three classes Ph1, Ph2, and Ph3.

Metabolite	Chemical class	*p* value	FDR	Tukey's HSD
*cis*-Aconitic acid	Misc	2.01*E* − 03	0.00015894	2-1, 3-1, 3-2
2,3,4,5-Tetrahydroxypentanoic acid 1,4-lactone	Ox	8.63*E* − 02	0.00026357	2-1, 3-1, 3-2
Erythronic acid	Ox	1.20*E* − 01	0.00026357	2-1, 3-1, 3-2
3-Hydroxybutyric acid	Misc	1.75*E* − 01	0.00026357	3-1, 3-2
3,4-Dihydroxybutyric acid	Misc	1.95*E* − 01	0.00026357	2-1, 3-2
Glucaric acid	Ox	2.00*E* − 01	0.00026357	2-1, 3-2
Unknown C	Misc	2.91*E* − 01	0.00029809	3-1, 3-2
Pseudouridine	Misc	3.02E − 01	0.00029809	3-1, 3-2
Erythritol	SR	3.98*E* − 01	0.00030706	3-1, 3-2
Gulonic acid	Ox	4.00*E* − 01	0.00030706	3-1, 3-2
Monosaccharide 1886	SR	4.28*E* − 01	0.00030706	3-1, 3-2
Pyroglutamic acid	Misc	5.02*E* − 01	0.00033075	2-1, 3-2
Arabitol	SR	5.44*E* − 01	0.00033075	3-1, 3-2
Fucose	Carb	5.94*E* − 01	0.00033536	3-1, 3-2
Ribitol	SR	6.53*E* − 01	0.00034397	2-1, 3-2
Tyrosine	AA	0.00011408	0.00056327	2-1, 3-2
Xylobiose	Carb	0.00016349	0.00075975	3-2
3,4,5-Trihydroxypentanoic acid	Misc	0.00023492	0.0010311	2-1, 3-2
Histidine	AA	0.00027084	0.0011261	2-1, 3-2
Gluconic acid	Ox	0.00040226	0.0015889	3-2
Serine	AA	0.00050574	0.0019025	2-1, 3-2
Lysine	AA	0.0005569	0.0019615	2-1, 3-2
Monosaccharide E	SR	0.00058834	0.0019615	3-2
Xylitol	SR	0.00059589	0.0019615	3-1, 3-2
Phenylalanine	AA	0.00089953	0.0028425	2-1, 3-2
Threonine	AA	0.00098451	0.0029914	2-1, 3-2
Quinolinic acid	Misc	0.0012588	0.003683	2-1, 3-2
4-Deoxythreonic acid	SR	0.0014511	0.0040942	3-2
Cystine	AA	0.0015954	0.004346	3-2
Succinic acid	Misc	0.0024091	0.0063441	3-2
Alanine	AA	0.0027785	0.0070807	2-1, 3-2
*N*-Acetylglucosamine	SR	0.0035822	0.0088435	3-2
Phosphate	Misc	0.0037469	0.0089697	3-2
Ribonic acid	Ox	0.0042205	0.0098065	3-2
2-Amino-6-hydroxy-7-methyl-7H-purine	Misc	0.0043539	0.0098274	3-1, 3-2
Hydroxyproline dipeptide	AA	0.0048016	0.010537	2-1, 3-2
Maltose	Carb	0.0050599	0.010804	2-1, 3-1
Glutamine	AA	0.0055744	0.011425	2-1, 3-2
Glycine, *N*-4-hydroxybenzoyl	Misc	0.0057479	0.011425	2-1, 3-2
Threonic acid	Ox	0.005785	0.011425	2-1, 3-2
2-O-Glycerol-galactopyranoside	SR	0.006708	0.012925	2-1, 3-2
Lactose	Carb	0.0077141	0.01451	2-1, 3-2
Ribose	Carb	0.0088787	0.016312	3-2
Citric acid	Misc	0.0092274	0.016567	3-2
Galactose	Carb	0.010113	0.017412	3-1
4-Hydroxyphenylacetic acid	Misc	0.010139	0.017412	2-1, 3-2
Glucose	Carb	0.012002	0.020173	2-1
Glycine, *N*-4-hydroxybenzoyl derivative	Misc	0.018028	0.029671	3-2
Uric acid	Misc	0.0197	0.031761	3-2
Threitol	SR	0.020579	0.032514	3-2
Galactitol	SR	0.021507	0.033314	2-1
Hippuric acid	Misc	0.023726	0.035603	3-2
Creatinine	Misc	0.023885	0.035603	2-1, 3-2
2,4-Dihydroxybutyric acid	Misc	0.026377	0.038588	3-2
2-Ketogluconic acid	Ox	0.027386	0.039336	3-2
Arabinose	Carb	0.028179	0.039753	3-1
Sedoheptulose	Carb	0.028971	0.040153	3-2
4-Deoxyerythronic acid	Ox	0.034187	0.046565	3-2

**Table 2:** Statistically significant (FDR < 0.05) metabolites from the *t*-test of the classes Ph2 versus Ph3.

Metabolite	Chemical class	*p* value	FDR	Ph2 versus Ph3
Gulonic acid	Ox	7.31*E* − 04	5.63*E* − 01	Down
Erythronic acid	Ox	3.00*E* − 02	7.03*E* − 01	Down
2,3,4,5-Tetrahydroxypentanoic acid 1.4-lactone	Ox	3,53E-03	7.03*E* − 01	Down
*cis*-Aconitic acid	Misc	4,44E-02	7.03*E* − 01	Down
Glucaric acid	Ox	4.76*E* − 03	7.03*E* − 01	Down
Pyroglutamic acid	Misc	5.48*E* − 02	7.03*E* − 01	Down
Ribitol	SR	9.12*E* − 02	9.12*E* − 01	Down
Arabitol	SR	9.47*E* − 02	9.12*E* − 01	Down
Fucose	Carb	1.07*E* − 01	9.14*E* − 02	Down
Unknown C	Misc	1.34*E* − 01	0.00010306	Down
Tyrosine	AA	1.52*E* − 01	0.0001065	Down
Erythritol	SR	1.87*E* − 01	0.00011968	Down
Pseudouridine	Misc	2.55*E* − 01	0.00015105	Down
3,4-Dihydroxybutyric acid	Misc	4.19*E* − 01	0.00023071	Down
Monosaccharide 1886	Carb	5.13*E* − 01	0.00026311	Down
Xylobiose	Carb	5.80*E* − 01	0.00027903	Down
Gluconic acid	Ox	6.60*E* − 01	0.00029457	Down
Histidine	AA	7.26*E* − 01	0.00029457	Down
4-Deoxythreonic acid	Ox	7.27*E* − 01	0.00029457	Down
3-Hydroxybutyric acid	Misc	9.00*E* − 01	0.00034512	Down
Phenylalanine	AA	0.00010095	0.00034512	Down
Monosaccharide E	Carb	0.0001043	0.00034512	Down
3,4,5-Trihydroxypentanoic acid	Ox	0.00010624	0.00034512	Down
Sedoheptulose	Carb	0.00010757	0.00034512	Down
Quinolinic acid	Misc	0.00012733	0.00039216	Down
Xylitol	SR	0.00017953	0.00053168	Down
Phosphate	Misc	0.00020953	0.00059755	Up
Lysine	AA	0.000373	0.0010074	Down
Succinic acid	Misc	0.00037941	0.0010074	Down
Ribose	Carb	0.00049401	0.001268	Down
Threonine	AA	0.00055591	0.0013069	Down
Cystine	AA	0.00056022	0.0013069	Down
2-Amino-6-hydroxy-7-methyl-7H-purine	Misc	0.00056835	0.0013069	Down
Serine	AA	0.00057708	0.0013069	Down
*N*-Acetylglucosamine	SR	0.00074989	0.0016498	Down
Citric acid	Misc	0.0009237	0.0019757	Down
Ribonic acid	Ox	0.0013077	0.0027213	Down
Glutamine	AA	0.0014072	0.0028515	Down
Lactose	Carb	0.0020445	0.0040365	Up
Glucose	Carb	0.0024816	0.004777	Down
Hydroxyproline dipeptide	AA	0.0028275	0.0053102	Down
Glycine, *N*-4-hydroxybenzoyl	Misc	0.0038577	0.0070724	Down
2-Ketogluconic acid	Ox	0.0045002	0.0080016	Down
2,4-Dihydroxybutyric acid	Misc	0.0045723	0.0080016	Down
Threonic acid	Ox	0.0047704	0.0081627	Down
4-Hydroxyphenylacetic acid	Misc	0.0051136	0.0085598	Down
Alanine	AA	0.0052474	0.0085968	Down
2-O-Glycerol-galactopyranoside	SR	0.0071983	0.011547	Down
Threitol	SR	0.0091347	0.014354	Down
Glycine, *N*-4-hydroxybenzoyl derivative	Misc	0.010305	0.015869	Down
Creatinine	Misc	0.012953	0.019557	Down
Uric acid	Misc	0.014046	0.020799	Up
3-Methylhistidine	AA	0.014579	0.02117	Down
Glyceromannoheptonic acid	Ox	0.014847	0.02117	Down
Hippuric acid	Misc	0.015776	0.022086	Down
Inositol	SR	0.016169	0.022232	Down
Arabinose	Carb	0.017089	0.023086	Down
4-Deoxyerythronic acid	Ox	0.017767	0.023587	Down
Ethanolamine	Misc	0.024206	0.031591	Down
Mannitol	SR	0.026258	0.033697	Down
